# Fungal-Mediated Multitrophic Interactions - Do Grass Endophytes in Diet Protect Voles from Predators?

**DOI:** 10.1371/journal.pone.0009845

**Published:** 2010-03-24

**Authors:** Susanna Saari, Janne Sundell, Otso Huitu, Marjo Helander, Elise Ketoja, Hannu Ylönen, Kari Saikkonen

**Affiliations:** 1 Plant Production Research, MTT Agrifood Research Finland, Jokioinen, Finland; 2 Department of Biology, University of Turku, Turku, Finland; 3 Department of Biological and Environmental Science, Konnevesi Research Station, University of Jyväskylä, Konnevesi, Finland; 4 Suonenjoki Research Unit, Finnish Forest Research Institute, Suonenjoki, Finland; 5 Services Unit, MTT Agrifood Research Finland, Jokioinen, Finland; 6 Lammi Biological Station, University of Helsinki, Lammi, Finland; Umea University, Sweden

## Abstract

Plant-associated micro-organisms such as mycotoxin-producing endophytes commonly have direct negative effects on herbivores. These effects may be carried over to natural enemies of the herbivores, but this has been rarely explored. We examined how feeding on *Neotyphodium* endophyte infected (E+) and endophyte free (E−) meadow ryegrass (*Scherodonus pratensis*) affects body mass, population size and mobility of sibling voles (*Microtus levis*), and whether the diet mediates the vulnerability of voles to least weasel (*Mustela nivalis nivalis*) predation. Because least weasels are known to be olfactory hunters, we also examined whether they are able to distinguish olfactory cues of voles fed on E+ and E− diets. Neither body mass of voles nor population size differed between diets. However, contrary to our prediction, least weasels preyed more often on voles fed with E− grass than on voles fed with E+ grass. The mobility of voles fed on E+ grass was reduced compared to voles fed on E− grass, but this effect was unrelated to risk of predation. Least weasels appeared unable to distinguish between excrement odours of voles between the two treatments. Our results suggest that consumption of endophytic grass is not directly deleterious to sibling voles. What's more, consumption of endophytes appears to be advantageous to voles by reducing risk of mammalian predation. Our study is thus the first to demonstrate an effect of plant-associated microbial symbionts on herbivore-predator interactions in vertebrate communities.

## Introduction

Although microbial interactions within and between trophic levels are ubiquitous, their roles have remained largely ignored in community scale studies [1]. Microbial symbionts have profound phenotypic effects on their hosts which may cascade upward through food webs [2]. For instance, plants host multiple symbiotic microbes, including mycorrhizal fungi, endophytic fungi and bacteria that may affect the performance of herbivores through symbiont-produced toxins which accumulate in herbivore tissue and thereby directly harm predators following ingestion of the prey. In addition, effects on higher trophic levels may manifest, e.g., via changes in herbivore densities, population dynamics, body size or behaviour of herbivores [3], [4].

One group of microbial symbionts that are known to affect multitrophic interactions are fungal grass-endophytes in the genus *Neotyphodium*. They are known to infect 20–30% of all grass species [5], forming systemic and asymptomatic infections throughout the aerial parts of the host plant, including the seeds, and thereby allowing vertical dispersal of the endophyte from one plant generation to another [6].

Grass-endophytes may have multifarious effects on herbivore communities [7]. Consumption of *Neotyphodium* endophyte origin mycotoxins has long been known to cause severe livestock disorders, including symptoms ranging from trembling to staggering and severe muscle spasms that cause animals to collapse [8], [9]. Also smaller vertebrate herbivores, such as rodents, are commonly negatively affected by endophyte ingestion. These effects include, e.g., decrease in population density [10], lowered body mass [11], increased toxicity-induced mortality [12] and suppression of reproduction and growth [13]. The alkaloids produced by endophytes may also have negative effects on the natural enemies of invertebrate herbivores [14], [15]. However, experimental studies on endophytes and their effects on higher trophic levels are still scarce [2], [16], and no study has examined the cascading effects of fungal symbionts of grasses on vertebrate food chains.

Here, we examined how feeding on endophyte (*Neotyphodium uncinatum* (Gains, Petrini & Schmidt) Glenn, Bacon, Price & Hanlin) infected (E+) or endophyte free (E−) meadow ryegrass (*Scherodonus pratensis* (Huds.) P. Beauv ex. *Lolium pratense*) affects body mass and population size of sibling voles (*Microtus levis* Miller ex *M. rossiaemeridionalis* Ognev) and whether the E+ diet influences the vulnerability of voles to predation by their most important natural enemy, the least weasel (*Mustela nivalis nivalis* L.). Because least weasels are known to be olfactory hunters [17], we also examined whether least weasels are able to distinguish olfactory cues of voles fed on E+ and E− diets. Based on previous studies we predicted that i) consumption of mycotoxic E+ grass has direct negative effects on voles and that ii) these effects influence the vulnerability of voles to their most important natural enemy, the least weasel (*Mustela nivalis nivalis* L.). Furthermore, if endophyte-containing diet affects the chemical composition of vole urine, as demonstrated by Huitu *et al.* (2008), we predict that iii) olfactory hunting least weasels might be able to discriminate and prefer the scent of weaker vole prey fed on E+ grass from those fed on E− grass.

## Results

**Figure 1:**
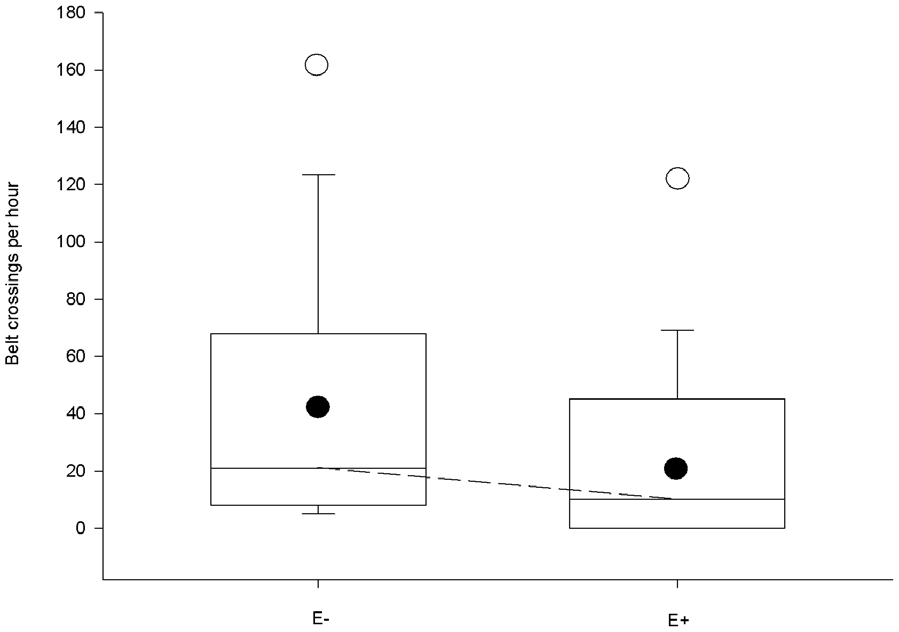
Mobility of voles. Differences in mobility of sibling voles as defined by numbers of belt crossings per hour for voles fed on endophyte infected (E+) or endophyte free (E−) grasses. The upper and lower boundaries of the box indicate the upper and lower quartiles, respectively. The horizontal line denotes the median. Vertical bars represent the tails of the distribution. Medians of the groups are connected with dotted line. Filled circles represent mean values. Mild outliers are marked with open circles. The number of replicates is 24.

Consumption of E+ grass did not have negative effects on vole population size or body mass. In the vole population experiment the estimated median difference in the minimum number of voles alive was only 0.5 individuals (95% CI for difference = −6 – +7; p = 1.0), in favour of the E− grass populations (means and standard deviations, voles alive: E+ 13.2±9.07, E− 13±5.15) In the vole body biomass experiment, females fed on E+ grass had on average 0.2 g (95% CI for difference = −1.0–+0.7; p = 0.66) lower and males 0.3 g (95% CI = −0.4–+1.1; p = 0.35) higher body mass compared to voles fed on E− grass (means and standard deviations, vole body mass: E+ female 21.32±3.10, E− female 21.49±2.75, E+ male 25.66±3.64 and E− male 25.31±3.66). Voles maintained on E+ grass exhibited lower mobility in the predation experiment than E− voles (estimated median difference in the activity of voles = 13.3 belt crossings/hour in favour of E− voles, 95% CI = 2.7–35.4; Fig. 1).

**Figure 2:**
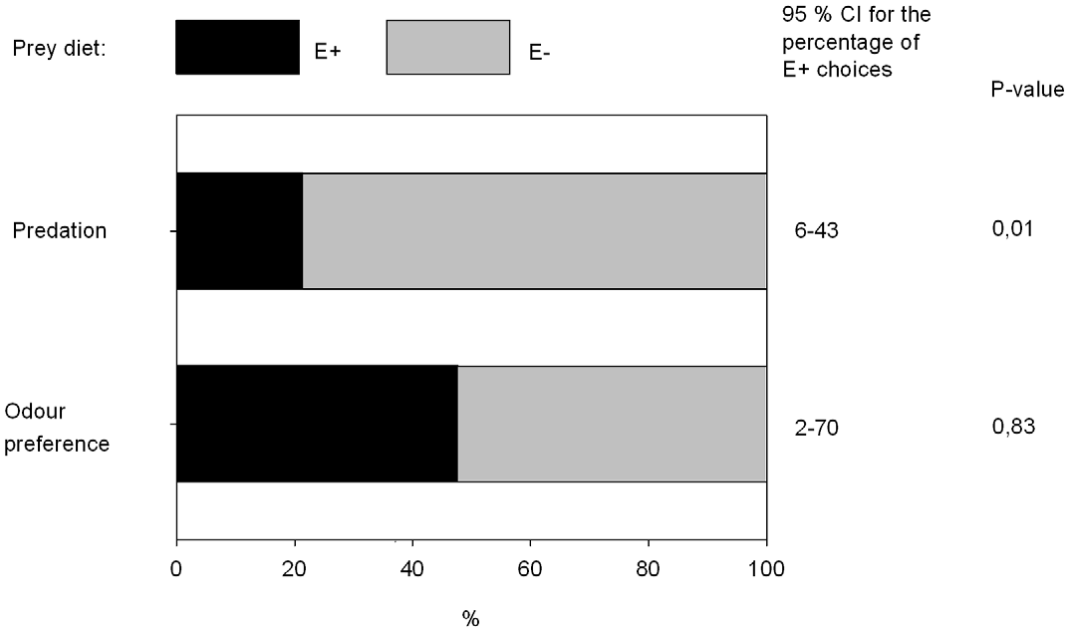
Predation and vole odour preference of weasel. The effects of endophyte infected (E+) and endophyte free (E−) grass diets on predation and vole odour preference of least weasel. Estimated percentages of captured voles (n = 17) and odour preference (n = 21).

Least weasels were more likely to capture voles fed on E− grass than voles fed on E+ grass (14 voles fed on E− captured versus three voles fed on E+ captured in the 17 successful trials; Fig. 2). However, activity of voles did not explain the susceptibility of voles to least weasel predation, nor did vole sex, length of the feeding period or body mass difference between the voles fed on E+ and E− diets in the beginning of the experiment (Table 1). Least weasels appeared unable to distinguish between the olfactory cues of voles fed on the two grass types; 10 least weasels chose the bedding of a vole fed on E+ grass and 11 chose the bedding of a vole fed on E− grass (Fig. 2).

**Table 1:** Results of fitting logistic regression models to the data of least weasel predation.

Explanatory variable	Coefficient 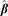	P-value for H_0_: 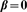	Odds Ratio 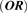 *)	95% CI for 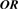 †)
Difference between E- and E+ voles in the number of belt crossings per hour ( = activity_E−_ − activity_E+_)	0.01	0.25	1.01	0.99–1.04
Sex of vole	−0.60	0.65	0.55	0.00–6.04
Length of the feeding period (days)	0.14	0.19	1.15	0.95–1.40
Difference between E− and E+ voles in weight ( = weight_E−_ − weight_E+_)	0.12	0.81	1.13	0.42–3.22

*)The estimated odds ratio 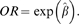 For quantitative explanatory variables 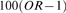 indicates the percent change in the odds of E+ vole being captured for each 1-unit increase in the explanatory variable. For example, for every one day increase in the length of the feeding period, the odds of E+ vole being captured increases by 15%. For categorical variable sex the 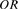 of 0.55 implies that for females the odds of E+ vole being captured is 0.55 times the odds for males, i.e. 45% lower.

†)The 95% confidence interval for the 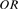 indicates the range of values within which the odds ratio from 95 of 100 similar studies would be expected to fall. The 95% CI also indicates the precision of the estimated 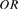.

## Discussion

Although E+ grasses are commonly thought to be chemically protected against herbivores [7], [16], our results with sibling voles and meadow fescue do not support this notion unanimously. Contrary to our predictions, we did not find negative effects of an E+ diet either on population size or body mass of sibling voles. It appears obvious that *Neotyphodium*-infected meadow ryegrass is not particularly toxic to sibling voles, at least within a time frame of a few months. Endophyte-infected meadow ryegrass has previously been shown to decrease body mass in a closely related vole species, the field vole (*Microtus agrestis* L.) in laboratory conditions [11]. It is therefore plausible that tolerance to loline mycotoxins varies among different vertebrate herbivore species. The discrepancy between this and earlier studies may also stem from variation in mycotoxin production, which is known to be dependent on environmental conditions [7], [16].

Contrary to our predictions, least weasels preyed more often on voles fed with E− grass than on voles fed with E+ grass. Voles that had consumed E− grass were also more mobile than voles that had consumed E+ grass. Although high mobility is often associated with increased predation risk [18], the degree of vole mobility was unrelated to the prey selection behaviour of least weasels in our experiment. Reduced mobility might be expected if mycotoxins had reduced the physiological well-being of voles to a point of apathy. However, this is not plausible in the light of our experiments, as voles did not lose body mass or show reduced population growth.

Voles exhibit an array of behaviours in their avoidance of predators. Many of these are related to mobility, for example fleeing and freezing [19]. The latter behaviour was frequently observed in encounters between E+ voles and weasels, so it is plausible that reduced mobility was related to freezing under predation risk. Furthermore, as weasels did not differentiate between odors of voles maintained on the different diets, we regard differences in the voles' antipredatory behaviour the most parsimonious explanation for the observed patterns in weasel prey selection. However, this reasoning is indicative at best, as unfortunately specifics of vole avoidance behaviour were not recorded. We also cannot conclude how vole mobility overall, regardless of treatment, was affected by the presence of predators, since mobility was not measured in the absence of predators in the system. Furhermore, freezing may have affect odour compounds in bedding and we are indeed unaware of whether the toxic compounds were transmitted to the urine.

### Conclusions

In our study we were able to demonstrate indirect positive effects of microbial plant symbionts on a vertebrate herbivore via reduced predation. Similar effects have been previously demonstrated with invertebrate predators and parasites as natural enemies in food webs where herbivores feed on endophyte infected plants [14], [15], [20]. In cases where species can tolerate mycotoxins produced by the endophyte and are less at risk of predation due to endophyte consumption, the net effect of the endophyte on the host grass will be negative. Therefore the traditional view of endophytes as defensive plant mutualists may not hold if a third trophic level is included. Thus, our results provide evidence that the nature of the relationship between grass endophytes and their hosts may range from mutualism to parasitism depending on the complexity of the food web.

## Materials and Methods

### Ethics statement

All procedures involving voles were carried out in accordance with the Act on the Use of Animals for Experimental Purposes established by the Ministry of Agriculture and Forestry, Finland. The study was approved and supervised by the Animal Experiment Committee of Finland (License number: STH393A).

### Species

Meadow ryegrass, the experimental plant species, is one of the most important forage grasses in Finland. It is a native grass species in Europe which occurs commonly outside of agronomic use in meadows, roadsides and wastelands in Finland [21]. Several widely used meadow ryegrass cultivars in Finland are commonly infected *Neotyphodium uncinatum* endophyte [22], which grows systemically in all parts of the host plant. *N. uncinatum* produces lolines which may cause variable responses in invertebrates and small vertebrates [7], [11] but the loline appears to be non-toxic to large mammal herbivores [23].

The sibling vole is a common and widely distributed species in southern and western Finland. The individuals used in the experiment were laboratory-born individuals, whose parents were trapped from natural populations in nearby fields of MTT Agrifood Research Finland, Jokioinen (60° 48′ 15″ N, 23° 29′ 10″ E), in autumn 2006. Prior to the experiment, voles were housed in ca. 60×40×40 cm^3^ cages (3–5 same sex individuals in a cage) and provided with *ad libitum* potatoes, water and twice a week with endophyte free fresh grass cut from the wild. Bedding was provided in the form of wood shavings and hay. Temperature in the laboratory was ca. 20°C and photoperiod 16 h light ∶ 8 h dark.

The least weasel is a common specialist predator of voles and their single most important source of mortality in natural populations [24], [25]. The least weasels used in the experiment were either first generation laboratory-born individuals from the Konnevesi Research Station of the University of Jyväskylä, Finland, or trapped from the wild but kept under similar conditions like lab-born ones for weeks before the experiment. Prior to the predation and olfactory experiments, the least weasels were housed individually in 60×80×60 cm^3^ cages in an outdoor shelter and provided with a rooster chick per day with one fasting day a week, and occasionally voles of the genera *Microtus* and *Myodes*. Bedding was provided in the form of wood shavings and hay.

### Vole population size

The experimental field was established to study the importance of endophytes on the population development of voles. Seeds (cultivar ‘Kasper’) were obtained in various seed lots from seed production farms via the Plant Production Inspection Centre, Loimaa, Finland. As samples, we stained 50 seeds per seed lot and examined them microscopically for endophyte status [26]. We chose two seed lots for the experiment: one uninfected (E−, 0% endophyte frequency) and the other infected (E+, 79% endophyte frequency). We sowed E+ and E− seeds in a field in five plot pairs (each plot 39×25 m^2^) so that we randomized E+ and E− treatments separately within each pair. The field was established in Jokioinen in May 2006. Each plot was surrounded with a sheet metal fence in order to keep the experimental voles inside and voles of natural populations and small mammal predators out of the experimental areas. The sheet metal was embedded 60 cm below ground while 60 cm remained above ground. Before sowing, the field was fertilized with cow manure (30 000 kg/ha) and again in June 2007 with a commercial fertilizer [16∶9∶22 (N ∶ P ∶ K) with micronutrients, Kemira, product number: 0647334]. All other plants except for meadow ryegrass were regularly rooted up from the field during the experiment.

Endophyte infection status of nine plant individuals per field plot was verified before the experiment using immunoplot assay to detect monoclonal antibodies specific to *Neotyphodium* (Phytoscreen Immunoplot Kit #ENDO7973, Agrinostics, Watkinsville, Georgia, USA). Alkaloid extracts of the plants were analysed [27], [28] and E+ plants were detected to harbor active endophyte infections producing loline alkaloids. 

Vole individuals (50 males and 50 females) were randomly selected from a larger pool of males and females, all of which were sexually mature and had a body mass of >20 g. Individuals were randomly assigned either to an E+ or an E− treatment. Thereafter, in August, five male and five female voles were released into each of the ten enclosures. Four and a half months later, which approximates the annual length of the reproductive period of voles in Finland, vole population sizes were estimated using Ugglan multiple live capture traps (Grahnab, Sweden). Five traps were placed in each enclosure under plywood shelter boxes to reduce exposure to the elements. The traps were baited with carrots and checked twice a day for three days. Population size was estimated for each enclosure with the minimum number alive –method [29].

### Vole body mass

Voles (72 individuals) were selected from a larger pool of males and females, all of which had a body mass of >16 g, and housed singly in cages. The selected voles were assigned into 36 pairs (15 female and 21 male pairs) based on similarity in body mass. At this stage, voles were provided *ad libitum* potato and water. On the following day, we assigned the vole individuals randomly either to an E+ or an E− diet treatment within each pair and recorded their body mass to the nearest 0.1 g with electronic scales. The mean differences in the body mass (body mass_E−_ − body mass_E+_) of females and males (and standard deviations of the differences) were 0.01 g (0.73 g) and 0.03 g (0.44 g), respectively. After this, we removed potatoes from the cages and provided the corresponding experimental diets, namely *ad libitum* fresh meadow ryegrass three times a day cut from respective E+ or E− plots in the field (see “*Vole population size*”). The body mass of the voles was again recorded when the voles had been maintained on the experimental diet for seven days.

### Prey preference of weasel

Prior to the predation experiment six female and 18 male vole pairs (one fed on E+ grass and one fed on E− grass within each pair) were fed with the experimental diets as in the body mass bioassay for 7–30 days. The length of the feeding period varied between vole pairs due to logistical reasons but was recorded for use a covariate in the analyses.

The experiment was conducted at the Konnevesi Research Station of the University of Jyväskylä (62° 37′ 40″ N, 26° 17′ 15″ E), Finland, in an experimental enclosure (10×10 m^2^) on a field naturally vegetated by meadow plant species (the field did not include meadow ryegrass). The enclosure was divided into six sectors separated by ca. 30 cm wide short-grass belts (cut 2 cm above ground surface) to enable monitoring of vole mobility. One experimental trial consisted of exposing both a vole fed on E+ and a vole fed on E− grass from the same feeding pair to least weasel predation in the enclosure. Thus, a replicate consisted of a pair of voles that were both the same sex, similar in weight in the beginning of the experiment and had been on the experimental diet for the same period of time. The voles were marked with fiber strips of different colors (15 cm long, 2 cm wide) bound around the pelvis, where it does not hinder movements, and released into the cut middle belt between the sectors of the enclosure. We randomized the strip color between voles fed on E+ and E− grass in every replicate. Simultaneously with releasing voles, we placed a least weasel in an Ugglan live trap in the same middle belt where the voles were released. The least weasel was released from the trap five minutes after releasing the voles. We observed and recorded the number of belt crossings of the voles in the enclosure until one of the voles was captured by a least weasel. A single trial lasted from 15 min to 9 hours. Seven out of the 24 trials had to be terminated for external reasons (darkness at night, heavy rain etc.) before the weasel had captured a vole. Different voles and least weasels were used in every replicate. After each trial we trapped the surviving vole (or both voles if a replicate had to be terminated) and removed them from the enclosure back to the laboratory.

### Odour preference of weasel

We studied whether least weasels, that are known to be olfactory hunters [19], distinguish and prefer olfactory cues of voles fed with E+ grass from voles fed with E− grass. As a source of odour we used urine- and faeces-soaked vole bedding material from cages of voles that had been feeding on either E− or E+ grass for the weasel predation experiments (see above). The collected bedding material was stored in air-tight plastic bags at −22°C for ca. three months. The bedding was thawed in the bags at room temperature before the experiment.

The vole odour experiment was carried out in a Y-maze arena [19], which consisted of three transparent Perspex (Perspex, Rotterdam, The Neatherlands) plastic tubes of 80 mm inside diameter and 80, 60 and 60 cm length, forming a Y. The weasel entered the 80 cm long tube and came to a bifurcation of ∼60°. There it had to choose to continue into either of the 60 cm tubes until it reached a target “nest box” containing vole odour at the end of the tube. The nest boxes were small laboratory cages (25×10×10 cm^3^) covered with a Perspex roof. The ends of the Perspex tubes were separated from the nest box by a Perspex door with 12 holes of 4-mm diameter to allow airflow.

In each nest box we placed a 2×10×10 cm^3^ wire mesh basket filled with vole bedding from either the E+ or the E− treatment. We randomized E+ and E− bedding from the same vole feeding pair to either end of the Y-maze prior each trial. Thus, we collected the E+ and E− bedding in each replicate from voles that had been on the experimental diet for the same amount of time. The entrance to the tube was a wooden box, in which we acclimatized the weasel for five minutes prior to the trial. The box was separated from the tube by a Perspex door that could be opened from outside the experimentation room with a monofilament line. The door had holes to allow airflow from the arena to the weasel box during the acclimatization period. Above the arena we mounted infrared light sources and a camera that was connected to a monitor in an adjacent room where the behavior of the weasel was monitored on screen. Altogether, we tested nine female and 12 male weasels. All weasels slowly approached the tube and selected one of the tubes at the bifurcation. The test ended when the weasel reached the end of either branch of the Y-tube and sniffed the holes at the door separating the tube from the nest box. After each trial we cleaned all parts of the arena with water and ca. 50% ethanol.

### Statistical methods

The response variables used in the statistical analyses and other details of the experiments are summarized in Table 2.

**Table 2:** Summary of the details of the experiments.

Experiment	Number of pairs	Number of voles Males Females		Criteria for pairing	Response variable in the statistical analysis
Vole population size	5	25	25	Same sex, approximately the same initial body mass.	Difference in the number of voles between E+ and E− in a enclosure pair after four and a half months.
Vole body mass	36	42	30	Same sex, approximately the same initial body mass.	Difference in body mass (g) between E+ and E− in a vole pair after feeding the voles seven days.
Prey preference of weasel	24	36	12	Same sex and length of the feeding period, approximately the same initial body mass.	Difference in the number of belt crossings per hour between E+ and E− in a vole pair
Prey preference of weasel	17*)	24	10	Same sex and length of the feeding period, approximately the same initial body mass.	E+ vole captured in a pair of voles fed on E+ and E− (yes, no)
Odour preference of weasel	21*)	42	0	Same sex and length of the feeding period, approximately the same initial body mass.	Bedding of E+ vole chosen in a E+ and E− bedding pair (yes, no)

*)Also the number of least weasels.

We analyzed the body mass of voles separately for females and males because variation in male body mass was higher than in females. The E+ and E− diet treatments were compared using a paired t-test and 95% confidence interval (CI) for the mean difference [30]. The analyses were performed by the MIXED procedure in version 9.1.3 of the SAS/STAT software. The rest of the data were analyzed through exact statistical methods because the data were small and non-normally distributed. We based the statistical analyses for least weasel predation on logistic regression models for binary data. Let 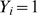 if E+ vole was captured and 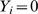 if E− vole was captured in pair 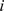. Each binary outcome variable 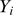 is assumed to have a Bernoulli distribution with parameter 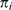, where 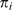 is the probability of E+ vole being captured in a pair 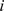. The value 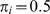 indicates an equal probability of capture for E+ and E− voles. When examining whether least weasels prefer E+ voles over E− voles as prey, the logistic regression model had the following form:
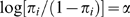
(1)where 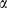 is a constant and the ratio 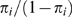 is the odds of E+ vole being captured in a pair 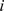. On fitting the model to the data, an estimate of 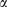, 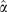, is obtained, and the estimated capture probability (proportion) 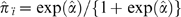. The 95% confidence limits for 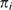 were computed similarly from the estimated limits of 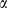. When examining the dependence of 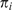 on the values 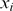 of each potential explanatory variable (one at a time), the model was of the following form:
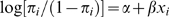
(2)where 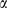 and 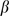 were the unknown parameters estimated by the data. We fitted the models by using the approach of conditional exact inference [31], and performed the analyses with version 8 of the LogXact software. The analysis of the binary choice data for vole odour was based on the corresponding model than the model (1) above. We tested the difference in the amount of captured voles per enclosure between E+ and E− grasses and also the difference in the belt crossings of voles/hour by using the exact Wilcoxon signed ranks test, and estimated the median difference and its 95% confidence interval (CI) by the Hodges-Lehmann procedure [32]. We performed the analysis with version 8 of the StatXact software.
